# 16S rRNA Methyltransferase RmtC in *Salmonella enterica* Serovar Virchow

**DOI:** 10.3201/eid1604.090736

**Published:** 2010-04

**Authors:** Katie L. Hopkins, Jose A. Escudero, Laura Hidalgo, Bruno Gonzalez-Zorn

**Affiliations:** Health Protection Agency Centre for Infections, London, UK (K.L. Hopkins); Universidad Complutense de Madrid, Madrid, Spain (J.A. Escudero, L. Hidalgo, B. Gonzalez-Zorn)

**Keywords:** Salmonella enterica, aminoglycosides, drug resistance, RmtC, methyltransferase, qnrB2, bacteria, dispatch

## Abstract

We screened *Salmonella* and *Escherichia coli* isolates, collected 2004–2008 in the United Kingdom, for 16S rRNA methyltransferases. *rmtC* was identified in *S. enterica* serovar Virchow isolates from clinical samples and food. All isolates were clonally related and bore the *rmtC* gene on the bacterial chromosome. Surveillance for and research on these resistance determinants are essential.

Aminoglycosides are used in treating a wide range of infections caused by both gram-negative and gram-positive bacteria and have been classified by the World Health Organization as critically important antimicrobial drugs in human medicine (*1*). They inhibit bacterial protein synthesis by binding irreversibly to the bacterial 16S ribosomal subunit, which thereby leads to cell death. Resistance to these antimicrobial agents usually results from production of aminoglycoside-modifying enzymes (such as acetyltransferases, phosphorylases, and adenyltransferases), reduced intracellular antimicrobial drug accumulation, or mutation of ribosomal proteins or rRNA. An additional mechanism, methylation of the aminoacyl site of 16S rRNA, confers high-level resistance to clinically important aminoglycosides such as amikacin, tobramycin, and gentamicin. Six types of 16S rRNA methyltransferase genes conferring resistance to these antimicrobial agents, *armA*, *rmtA*, *rmtB*, *rmtC*, *rmtD*, and *npmA*, have been identified (*2*,*3*). *armA* and *rmtB* are spread in enterobacteria worldwide, and the presence of other methyltransferase genes have not previously been reported in Europe (*3*). With the exceptions of *armA* and *rmtB* in porcine *Escherichia coli* from Spain and the People’s Republic of China, respectively (*4*,*5*), all methyltransferase genes described have been identified in human clinical samples, for which a possible role for food in transmission of these determinants remains largely unknown. Despite large surveys performed to identify 16S rRNA methyltransferases, the *rmtC* gene has been detected in only 2 *Proteus mirabilis* clinical isolates from Japan and Australia in 2006 and 2008, respectively (*3*,*6*,*7*). In this study, 81,632 *Salmonella* and 10,700 *Escherichia coli* isolates obtained from the Health Protection Agency (HPA) Centre for Infections culture collection (isolated from January 2004 through December 2008) were screened for the presence of 16S rRNA methyltransferases.

## The Study

*Salmonella enterica* (56 isolates) and *Escherichia coli* (24 isolates) were selected from the HPA collection based on their resistance to amikacin (breakpoint concentration routinely used in HPA *Salmonella* Reference Unit = 4 µg/mL). Because 16S rRNA methyltransferases confer high-level resistance to amikacin, 13 *S. enterica* isolates were selected on the basis of ability to grow on Isosensitest agar containing 500 µg/mL amikacin, whereas none of the *E. coli* isolates grew under these conditions. All isolates belonged to serotype Virchow. Further antimicrobial susceptibility testing by microdilution by using dehydrated Sensititer plates following the CLSI guidelines confirmed high-level resistance to 4,6-disubstituted 2-deoxystreptamines (Table 1). PCR screening of the 13 isolates for *armA*, *rmtA*, *rmtB*, *rmtC*, and *rmtD* (*8*) identified *rmtC*. Nucleotide sequencing of the amplicons confirmed an *rmtC* gene with 100% identity with those originally identified in *Proteus mirabilis* strain ARS68 isolated from an inpatient in Japan (*6*) and *P. mirabilis* strain JIE273 from Australia (*7*). To our knowledge, this is the third report of *rmtC*-bearing bacteria. Class one integrons were amplified (*9*), and sequenced. Isolates resistant to neomycin bore the *aac(6′)-Ib* gene cassette, whereas the *dfrA1* gene was responsible for resistance to trimethoprim.

**Table 1:** Phage types for *Salmonella enterica* ser. Virchow isolates bearing *rmtC* and MICs of selected antimicrobial agents*

Isolate	Phage type	GEN	KAN	AMK	TOB	ARB	NEO	TMP	CPX	AMP
HO 5164 0340	ND	>512	>512	>512	>512	>512	64	<0.5	0.5	1
HO 5366 0426	30	>512	>512	>512	>512	>512	2	>32	0.25	<0.05
HO 6018 0151	30	>512	>512	>512	>512	>512	2	>32	0.25	1
HO 6316 0322	30	>512	>512	>512	>512	>512	32	<0.5	0.25	1
HO 6398 0463	30	>512	>512	>512	>512	>512	32	<0.5	0.25	1
HO 7078 0136	30	>512	>512	>512	>512	>512	2	>32	0.25	<0.05
HO 7310 0210	31	>512	>512	>512	>512	>512	4	>32	0.25	1
HO 7468 0335	25	>512	>512	>512	>512	>512	2	>32	0.5	1
HO 7474 0467	25	>512	>512	>512	>512	>512	4	>32	0.25	<0.05
HO 7496 0137	25	>512	>512	>512	>512	>512	2	>32	0.25	1
HO 7512 0259	25	>512	>512	>512	>512	>512	4	>32	0.25	1
HO 8354 0857	25	>512	>512	>512	>512	>512	4	>32	0.25	<0.05
HO 8512 0713	25	>512	>512	>512	>512	>512	4	>32	0.25	1

*MICs are given in µg/mL. GEN, gentamicin; KAN, kanamycin; AMK, amikacin; TOB, tobramycin; ARB, arbekacin; NEO, neomycin; TMP, trimethoprim; CPX, ciprofloxacin; AMP, ampicillin; ND, not determined.

**Figure 1:**
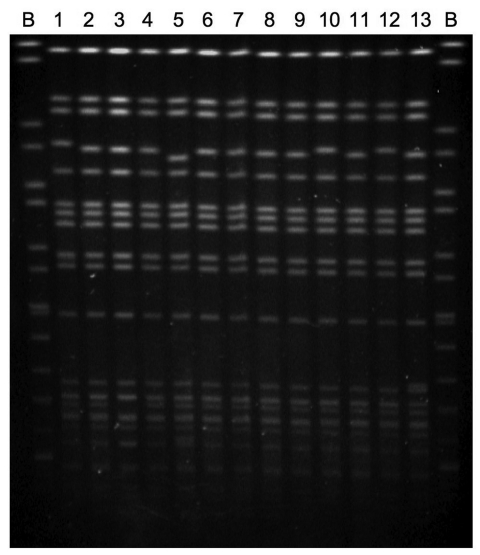
Pulsed-field gel electrophoresis patterns of *rmtC*-positive *Salmonella enterica* serovar Virchow isolates. Lanes: B, *S.* Braenderup H9812 size standard; 1, H0 5164 0340; 2, H0 5366 0426; 3, H0 6018 0151; 4, H0 6136 0322; 5, H0 6398 0463; 6, H0 7078 0136; 7, H0 7310 0210; 8, H0 7468 0335; 9, H0 7474 0467; 10, H0 7496 0137; 11, H0 7512 0259; 12, H0 8354 0857; and 13, H0 8512 0713**.**

**Figure 2:**
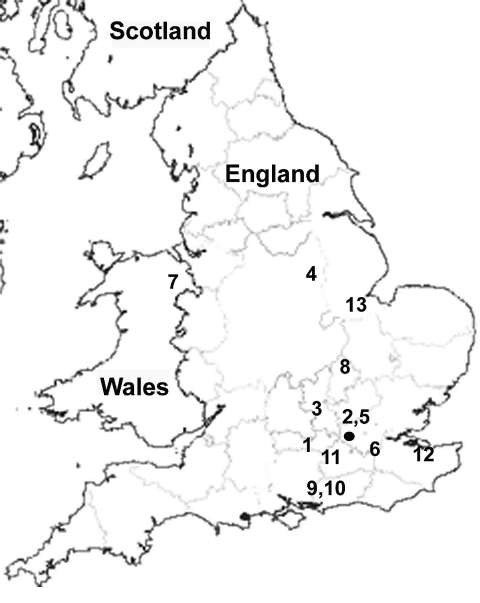
Map of United Kingdom showing geographic location of the 13 *Salmonella* *enterica* serovar Virchow isolates bearing *rmtC*. Each number represents 1 isolate in chronologic order of isolation as shown in Table 2.

Twelve of the 13 *S. enterica* strains were originally isolated over a 4-year period from patients with clinical infection; 1 strain was obtained from frozen produce. Seven of 12 strains were obtained from patients with histories of foreign travel; 4 of the 7 patients had reported recent travel to India (Table 2). *P. mirabilis* strain JIE273 was also isolated from a patient who had recently returned from India (*7*). Investigations to ascertain the presence of *rmtC* genes in India are under way. To identify a possible link between the isolates, chromosomal DNA was embedded in agarose plugs prepared according to the pulsed-field gel electrophoresis (PFGE) protocol of PulseNet Europe (*10*). PFGE patterns showed only 1–2-band differences (Figure 1) and correlated with phage typing data (Table 1). All clinical isolates were recovered from feces, except a blood isolate recovered from a patient with invasive salmonellosis (Table 2). The temporal and geographic distribution of the isolates suggested independent acquisition of infections in most cases and possibly epidemiologically linked cases, e.g., strains 9 and 10 (Table 2; Figure 2).

**Table 2:** Epidemiologic information from *rmtC-*positive *Salmonella enterica* serovar Virchow isolates, United Kingdom, 2004–2008*

Isolate	Date received	Map no.	Location	Sample type	Symptoms	Travel history
HO 5164 0340	2005 Apr 20	1	Reading	Feces	ND	ND
HO 5366 0426	2005 Sept 8	2	London	Feces	Diarrhea	Unknown destination
HO 6018 0151	2006 Jan 6	3	Wexham	Feces	ND	ND
HO 6316 0322	2006 Aug 3	4	Nottinghamshire	Feces	Diarrhea	No recent travel
HO 6398 0463	2006 Sept 29	5	London	Blood	Fever and diarrhea	India
HO 7078 0136	2007 Feb 16	6	Orpington	Feces	Diarrhea	India
HO 7310 0210	2007 July 30	7	Wrexham	Feces	Diarrhea	Unknown destination
HO 7468 0335	2007 Nov 16	8	Bedford	Feces	Enteritis	India
HO 7474 0467	2007 Nov 21	9	West Sussex	Feces	Diarrhea	ND
HO 7496 0137	2007 Dec 6	10	West Sussex	Feces	Diarrhea	ND
HO 7512 0259	2007 Dec 18	11	Surrey	Feces	Diarrhea	India
HO 8354 0857	2008 Aug 27	12	Kent	ND	Diarrhea	Unknown destination
HO 8512 0713	2008 Dec 16	13	Spalding	Food	NA	NA

*ND, not determined; NA, not applicable.

PCR with primers ISEcp1R-F and *rmtC*-down (*7*) showed that the *rmtC* gene and immediate upstream sequences (GenBank accession nos. FJ984623–FJ984634 for human isolates and GQ131574 for the food isolate) were identical to those previously identified in *P. mirabilis* (*6*,*7*), in which IS*Ecp1* has been shown to play a role in the expression and transposition of the *rmtC* gene (*11*). However, the complete IS*Ecp1* element could not be amplified by using primers IS*Ecp1* 5′ and IS*Ecp1* reverse, which suggests either partial deletion of this element or involvement of a different IS*Ecp1*-like element in spread of *rmtC* in *Salmonella* (*6**,**12*). Attempts to isolate *rmtC* by conjugal transfer to rifampin-resistant *E. coli* 20R764 were unsuccessful, as was electroporation into *E. coli* LMG194 and ElectroMAX DH10B cells (both Invitrogen, Paisley, UK) by using plasmid preparations. An ≈100-kb *rmtC*-bearing plasmid was previously transferred from *P. mirabilis* ARS68 by electroporation but could not be mobilized by conjugation (*6*), and attempts to transfer the *rmtC* plasmid from *P. mirabilis* JIE273 by electroporation and conjugation failed (*7*). This finding contrasts with some qualities of the other methyltransferases, such as *armA* and *rmtB*, which are mostly located on conjugative plasmids (*8**,**13*).

The location of the *rmtC* gene was determined with PFGE by using I-*Ceu*I nuclease treatment. Agarose plugs were digested with 9.5 U I-*Ceu*I nuclease (New England Biolabs, Beverly, MA, USA). Separated DNA fragments were transferred onto a nylon membrane (GE Healthcare, Madrid, Spain) and hybridized with 16S rDNA and *rmtC* probes labeled with DIG-11-dUTP. Hybridization, labeling, and detection were performed according to the manufacturer’s recommendations (Roche Applied Science, Mannheim, Germany). A DNA band hybridized with both probes, showing that the *rmtC* gene was located on the chromosome. Results of hybridization of plasmid extractions (Plasmid Midi kit; QIAGEN, Inc., Chatworth, CA, USA) with the *rmtC* probe were negative (data not shown).

## Conclusions

We describe the occurrence of 16S rRNA methyltransferase *rmtC* in *Salmonella* isolates and the *rmtC* gene in Europe. We also report that a producer of 16S rRNA methyltransferase was isolated from food.

The overall isolation frequency of 16S rRNA methyltransferase–producing *S. enterica* is low (13/81,632 strains) in the United Kingdom, and these genes were absent in *E. coli*. However, spread of multidrug-resistant isolates that express 16S rRNA methyltransferases, amplified by the association of these genes with the IS*Ecp1* element, raises clinical concern that further spread is likely. Ongoing surveillance of 16S rRNA methyltransferases in isolates found in food products and in humans and animals is crucial to delay the spread of resistance to these classes of antimicrobial agents.

## Addendum

While this manuscript was under revision, an *S. enterica* ser. Virchow isolate bearing the *rmtC* gene isolated from a child with a history of travel to India was reported in the United States (*14*).
